# Improvement in Antipsychotic-Related Sexual Dysfunction After Switching to Cariprazine: A Prospective Real-World Study

**DOI:** 10.3390/jcm15093320

**Published:** 2026-04-27

**Authors:** Ángel L. Montejo, Juan C. Fiorini, Pedro Megía, Rubén Ochoa, Belén Arribas, Marc Peraire, Iván Echeverria, Eladio Aparicio, Llanyra García-Ullán

**Affiliations:** 1 Psychiatry Service, Hospital Clínico Universitario de Salamanca, 37007 Salamanca, Spain; llanyra@usal.es; 2 Institute of Biomedical Research of Salamanca (IBSAL), 37007 Salamanca, Spain; 3 Faculty of Nursing, University of Salamanca, 37007 Salamanca, Spain; 4 Department of Psychiatry, Complejo Asistencial de Palencia, 34005 Palencia, Spain; jcfiorini@saludcastillayleon.es (J.C.F.); pmegia@saludcastillayleon.es (P.M.); rochoa@saludcastillayleon.es (R.O.); 5 Department of Psychiatry, Hospital Universitario de Valladolid, 47005 Valladolid, Spain; barribas@saludcastillayleon.es; 6 Department of Psychiatry, Consorcio Hospitalario Provincial de Castellón, 12002 Castellón de la Plana, Spain; mperaire@hotmail.com (M.P.); iechevarria@hospitalprovincial.es (I.E.); 7 TXP Research Group, Universidad Cardenal Herrera-CEU, CEU Universities, 46115 Castellón de la Plana, Spain; 8 Pre-Departmental Medicine Unit, Universitat Jaume I, 12006 Castellón de la Plana, Spain; 9 Department of Psychiatry, Hospital Clínico Universitario Virgen de la Arrixaca, 30120 Murcia, Spain; eladio.aparicio@carm.es; 10 Faculty of Medicine, University of Salamanca, 37007 Salamanca, Spain

**Keywords:** antipsychotic-induced sexual dysfunction, cariprazine, prolactin-sparing antipsychotics, schizophrenia

## Abstract

**Background**: Sexual dysfunction is a frequent adverse effect of antipsychotic treatment and a major contributor to poor adherence and reduced quality of life. Evidence regarding the impact of switching to prolactin-sparing antipsychotics in routine clinical practice remains limited. This study evaluated changes in sexual function following initiation or switch to cariprazine in real-world patients with schizophrenia spectrum disorders. **Methods**: In this prospective observational study, adult outpatients were either initiated on cariprazine de novo (Group A) or switched from a previous antipsychotic due to clinically significant sexual dysfunction (Group B). Sexual function was assessed using the Psychotropic-Related Sexual Dysfunction Questionnaire (SALSEX) at baseline and Month 3. Secondary measures included serum prolactin levels and Brief Psychiatric Rating Scale (BPRS) an CGI scores. Effect sizes were calculated using Cohen’s d. **Results**: Forty-two patients were included (Group A: *n* = 14; Group B: *n* = 28). In Group B, mean SALSEX total scores significantly decreased from 8.04 ± 2.76 to 2.41 ± 2.06 (Δ = −5.63; *p* < 0.001; d = 2.27). Prolactin levels also significantly decreased after switching (*p* = 0.012). In Group A, SALSEX scores showed a statistically significant but clinically modest reduction (2.79 ± 2.01 to 1.23 ± 1.24; *p* = 0.023; d = 0.93), with no evidence of treatment-emergent sexual dysfunction. Improvements in sexual function were not associated with changes either in BPRS or CGI scores, baseline symptom severity, sex, or testosterone levels. **Conclusions**: Switching to cariprazine in patients with antipsychotic-related sexual dysfunction was associated with large and clinically meaningful improvement in sexual function in routine practice. The effect appeared independent of overall symptom improvement and endocrine normalization thresholds, supporting the clinical value of prolactin-sparing switching strategies.

## 1. Introduction

Sexual dysfunction (SD) is one of the most frequent, persistent, and clinically relevant adverse effects of antipsychotic treatment. The high prevalence of sexual dysfunction observed in patients with schizophrenia is consistent with findings from recent systematic reviews and meta-analyses conducted across diverse populations and both sexes [1,2]. Sexual dysfunction in schizophrenia affects not only adults but also adolescents and young individuals, highlighting broader unmet needs in sexual and reproductive health across the lifespan [3].

Although spontaneous reporting rates are typically below 20%, structured assessments consistently demonstrate prevalence rates exceeding 60–70% among sexually active patients receiving antipsychotics [4,5,6]. These findings are consistent with systematic reviews confirming the high burden of sexual dysfunction in schizophrenia spectrum disorders under antipsychotic treatment [7]. This discrepancy highlights a systematic under-recognition of sexual adverse effects in routine clinical practice.

Sexual dysfunction in psychotic disorders is multifactorial. Illness-related factors, negative symptoms, comorbid medical conditions, and lifestyle variables contribute to impaired sexual functioning. However, antipsychotic treatment—particularly dopamine D2 receptor antagonism and prolactin elevation—plays a central mechanistic role [8,9,10]. Hyperprolactinemia disrupts the hypothalamic–pituitary–gonadal axis, reduces gonadal hormones, and may lead to decreased libido, arousal impairment, orgasmic dysfunction, menstrual disturbances, and erectile dysfunction [9,10]. Nonetheless, prolactin-independent mechanisms—including serotonergic, adrenergic, cholinergic, and nitric oxide pathways—also contribute [5,11].

From a clinical standpoint, antipsychotic-induced sexual dysfunction is strongly associated with reduced quality of life, impaired intimate relationships, and, critically, poor adherence. Treatment discontinuation rates attributable to sexual adverse effects have been estimated at over 35% in men and approximately 20% in women receiving long-term antipsychotic therapy [5,12,13]. Despite these consequences, sexual health remains insufficiently addressed in psychiatric consultations.

Structured assessment significantly improves detection. The Psychotropic-Related Sexual Dysfunction Questionnaire (PRSexDQ-SALSEX) is a validated, brief instrument specifically developed for psychopharmacological settings [14,15].

Comparative evidence demonstrates marked variability among antipsychotics. Risperidone and paliperidone exhibit high prolactin liability and elevated rates of sexual dysfunction, whereas quetiapine, clozapine, ziprasidone, and partial dopamine agonists generally show lower prolactin elevation and improved sexual tolerability profiles [8,10].

Cariprazine is a dopamine D3/D2 partial agonist with preferential D3 receptor affinity and a prolactin-sparing pharmacological profile demonstrated in pooled analyses from schizophrenia and bipolar disorder trials [16,17,18,19]. However, available evidence regarding sexual functioning with cariprazine is largely indirect, derived from adverse event reporting or prolactin data in registration trials, rather than from studies using validated sexual-function instruments.

Switching to prolactin-sparing agents or adding partial agonists such as aripiprazole has shown efficacy in reducing hyperprolactinemia-related sexual dysfunction in prospective and observational studies [20,21,22,23]. To date, no prospective real-world studies have systematically evaluated sexual dysfunction outcomes after switching to cariprazine in patients with poorly tolerated antipsychotic-induced sexual dysfunction using a validated domain-based scale. Given the high clinical relevance of this adverse effect and its implications for adherence and long-term outcomes, direct evaluation is warranted.

The present study (SALSEX V) was therefore designed as a pragmatic, prospective, real-world investigation with two distinct cohorts: (A) patients initiating cariprazine de novo, and (B) patients switched to cariprazine due to poorly tolerated sexual dysfunction attributed to a prior antipsychotic. The primary objective was to evaluate longitudinal changes in SALSEX total and domain scores over three months. Secondary objectives included endocrine outcomes (prolactin and gonadal hormones), psychiatric stability, and treatment persistence.

By integrating structured sexual health assessment into routine antipsychotic management, this study aims to contribute clinically actionable real-world evidence to support patient-centered treatment optimization.

## 2. Materials and Methods

### 2.1. Study Population and Eligibility Criteria

The study population consisted of adult outpatients diagnosed with schizophrenia spectrum disorders or other psychotic disorders according to DSM-5 criteria and treated in routine clinical practice. Patients were recruited from real-world psychiatric outpatient settings and were either initiating cariprazine de novo (Group A) or switching to cariprazine due to clinically significant and poorly tolerated antipsychotic-induced sexual dysfunction (Group B).

A total of 42 patients were included in the study: 14 in Group A (de novo initiation) and 28 in Group B (switch due to sexual dysfunction). Overall, 26 participants were men and 16 were women. The mean age of the total sample was 39.8 ± 10.6 years. Among patients presenting with clinically significant sexual dysfunction at baseline (Group B), the mean age was 41.2 ± 9.8 years, with 18 men and 10 women affected.

Inclusion criteria were: (1) age ≥ 18 years; (2) confirmed DSM-5 diagnosis of a psychotic disorder; (3) clinical stability at the time of inclusion, defined as absence of acute psychotic exacerbation and no need for hospitalization; (4) stable psychiatric treatment during the previous four weeks; (5) documented sexual activity prior to the onset of current sexual dysfunction; and (6) capacity to understand and complete the SALSEX questionnaire.

For the switch group (Group B), additional inclusion criteria required the presence of clinically relevant sexual dysfunction temporally associated with previous antipsychotic treatment and considered by the treating psychiatrist to be poorly tolerated.

Exclusion criteria included: (1) use of concomitant medications known to significantly affect sexual function (e.g., antidepressants with high sexual side-effect burden, hormonal therapies not clinically indicated for hypogonadism, phosphodiesterase-5 inhibitors initiated during the study period); (2) uncontrolled endocrine disorders; (3) severe medical comorbidities affecting sexual function (e.g., advanced cardiovascular disease); (4) substance use disorder not in remission; (5) pregnancy or breastfeeding; and (6) inability to provide informed consent.

These criteria were applied to ensure that changes in sexual function could be reasonably attributed to antipsychotic treatment modification rather than confounding pharmacological or medical factors, while maintaining the pragmatic and naturalistic nature of the study.

### 2.2. Design and Setting

Prospective observational cohort with three visits: baseline, month 1, and month 3 (final). Participants were recruited from five psychiatric services in Spain, including the Psychiatry Departments of the University Hospital of Salamanca, Hospital Río Carrión (Palencia), and collaborating psychiatric services in Valladolid, Castellón, and Murcia. All investigators were consultant psychiatrists with clinical experience in the management of psychotic disorders and the assessment of psychotropic-related adverse effects. Recruitment was conducted in routine clinical practice settings, where enrollment in prospective observational studies addressing sexual dysfunction can be challenging due to underreporting of sexual adverse effects and the sensitive nature of the topic.

### 2.3. Outcomes

Primary: SALSEX total (0–15) and five domain items (0–3): (1) decreased libido; (2) delayed orgasm; (3) anorgasmia; (4) arousal impairment (erection/lubrication); (5) tolerability/distress [5,6]. Clinically relevant sexual dysfunction was operationally defined as a SALSEX total score ≥5 or the presence of at least moderate severity (score ≥ 2) in one or more domains.

Secondary: prolactin and gonadal hormones (endocrine testing was performed according to routine clinical practice reflecting real-world conditions), Brief Psychiatric Rating Scale (BPRS), Clinical Global Impression (CGI) measures, cariprazine dose and persistence.

Hormonal determinations (prolactin, LH, FSH, and testosterone) were performed as part of routine clinical laboratory assessments at each participating center. Blood samples were obtained under standardized morning conditions. Assays were conducted using validated immunoassay platforms routinely employed in hospital laboratories. Given the naturalistic design of the study, specific assay kits and calibration procedures were not centralized but followed standard clinical laboratory quality control protocols.

### 2.4. Statistics

Complete-case paired analyses for each outcome; mean ± SD (and prolactin median [IQR]). Paired *t*-tests for baseline → month 3 change; α = 0.05. Between-group comparisons are descriptive (indication-driven cohorts). Paired *t*-tests were selected due to the within-subject design comparing two time points (baseline and Month 3). Given the sample size and study design, ANOVA-based approaches were not considered necessary.

All analyses were conducted using SPSS (version 26.0; IBM Corp., Armonk, NY, USA). Descriptive statistics are presented as mean ± standard deviation (SD) for continuous variables and as frequencies and percentages for categorical variables. Normality of continuous variables was assessed using the Shapiro–Wilk test and visual inspection of histograms.

Within-group longitudinal changes in SALSEX total score and individual item scores from baseline to month 1 and month 3 were evaluated using paired Student’s *t*-tests or Wilcoxon signed-rank tests when normality assumptions were not met. Effect sizes were calculated using Cohen’s d for paired comparisons.

Between-group comparisons at baseline were explored using independent-samples *t*-tests or Mann–Whitney U tests for continuous variables and chi-square tests for categorical variables. Given the observational design and indication-driven group allocation, between-group analyses were considered exploratory.

Sex-stratified analyses were performed to explore potential differences in sexual dysfunction trajectories between male and female patients. Missing data were handled using complete-case analysis. All statistical tests were two-tailed, and statistical significance was set at *p* < 0.05.

## 3. Results

### 3.1. Baseline Characteristics

A total of 42 patients were included (Group A: *n* = 14; Group B: *n* = 28). Baseline characteristics are summarized in Table 1. Patients in the switch group exhibited significantly higher baseline sexual dysfunction, with mean SALSEX scores of 8.04 ± 2.76 compared to 2.79 ± 2.01 in the de novo group, reflecting the indication for treatment change. Baseline prolactin levels were also numerically higher in the switch group (42.07 ± 59.22 ng/mL vs. 14.89 ± 14.12 ng/mL), consistent with prior exposure to prolactin-elevating antipsychotics.

The overall sample included 26 men and 16 women, with a mean age of 39.8 ± 10.6 years. The de novo group showed higher baseline psychotic severity (CGI-S), reflecting initiation during active symptom phases, whereas the switch group was clinically more stable.

### 3.2. Effects of Cariprazine on Sexual Function

**Figure 1:**
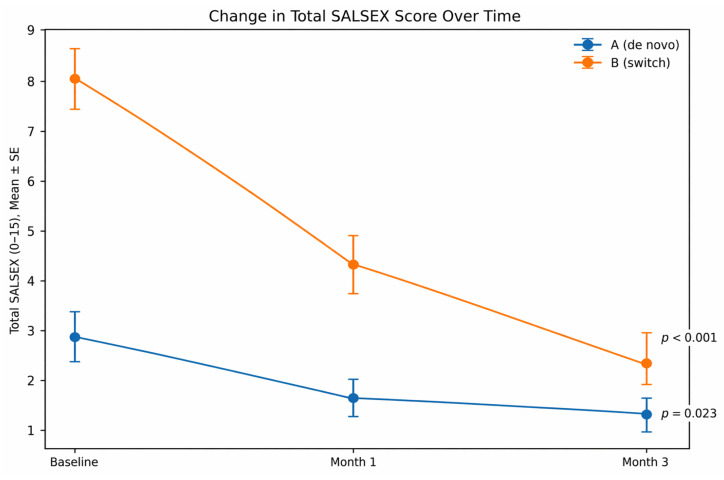
Longitudinal trajectories of SALSEX total score by group. SALSEX total Score from 0 (no dysfunction) to 15 (highest sexual dysfunction). Statistical comparisons were performed using paired *t*-tests. Error bars represent standard deviation.

**Figure 2:**
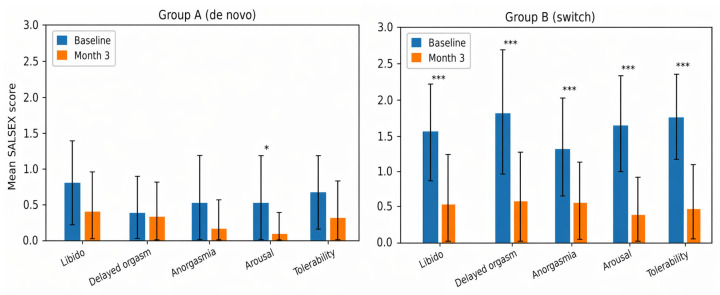
Change in SALSEX item scores from baseline to Month 3 by treatment group. (**Left panel**): Group A (de novo initiation). (**Right panel**): Group B (switch due to sexual dysfunction). Each domain is presented separately. Bars represent mean values and error bars indicate standard deviation. * *p* < 0.05; *** *p* < 0.001 (paired *t*-tests).

Changes in sexual function are presented in Table 2 and Figure 1 and Figure 2.

In the switch group, cariprazine treatment was associated with a large and clinically meaningful reduction in SALSEX total score, from 8.04 ± 2.76 at baseline to 2.41 ± 2.06 at Month 3 (Δ = −5.63; *p* < 0.001; d = 2.27). Improvement was already evident at Month 1 and progressed over time.

This reduction reflected a consistent improvement across all SALSEX domains (Table 3), with the largest effects observed in decreased libido, arousal impairment, and tolerability. Orgasm-related domains improved more gradually.

In the de novo group, SALSEX scores decreased modestly (2.79 ± 2.01 to 1.23 ± 1.24; *p* = 0.023), with no evidence of treatment-emergent sexual dysfunction.

No patient experienced clinically meaningful worsening of sexual function, and improvement was consistent across individuals.

### 3.3. Changes in Prolactin and Hormonal Parameters

**Figure 3:**
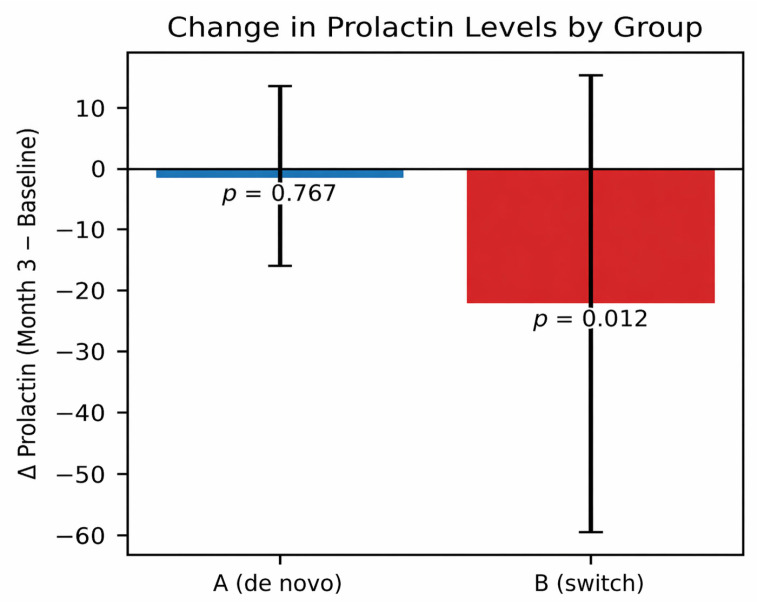
Change in prolactin from baseline to month 3 by group.

Hormonal outcomes are summarized in Table 4 and Figure 3.

In the switch group, prolactin levels significantly decreased from 42.07 ng/mL to 29.52 ng/mL (*p* = 0.012). In contrast, no significant change was observed in the de novo group.

No significant changes were detected in FSH, LH, or testosterone levels in either group.

Exploratory analyses did not show a significant correlation between prolactin reduction and improvement in sexual function, suggesting that additional mechanisms may contribute to the observed clinical effects.

### 3.4. Treatment Persistence and Clinical Outcomes

Treatment outcomes are summarized in Table 5 and Table 6.

Treatment persistence was high in both groups. In the de novo group, 13 of 14 patients completed the study. In the switch group, 22 patients remained on treatment, with minimal discontinuations unrelated to sexual function.

Psychiatric outcomes improved significantly in both groups, as reflected by reductions in BPRS scores. Importantly, changes in sexual function were not associated with changes in psychiatric symptom severity, supporting an independent pharmacological effect.

No cases of psychiatric destabilization or discontinuation due to sexual adverse effects were observed.

### 3.5. Sex Differences and Exploratory Analyses

Sex-stratified analyses showed comparable improvements in sexual function in both men and women. No significant sex differences were observed in SALSEX score reduction.

Testosterone levels remained stable in male patients, indicating that improvements in sexual function were not mediated by gonadal hormonal changes.

Exploratory analyses did not identify significant associations between sexual improvement and demographic variables, baseline severity, or psychiatric outcomes.

Overall, sexual improvement following switching to cariprazine was consistent and independent of sex, hormonal variation, or symptom change.

## 4. Discussion

The observed improvements in sexual function and prolactin levels are consistent with previous evidence on prolactin-sparing antipsychotics and partial dopamine agonists. Prior studies have demonstrated that switching from prolactin-elevating agents such as risperidone to agents with partial D2 agonism results in both endocrine normalization and clinically meaningful improvement in sexual functioning. The magnitude of effect observed in the present study is comparable to that reported with aripiprazole and other prolactin-sparing strategies, supporting the generalizability of this pharmacological approach.

Sexual dysfunction (SD) is one of the most frequent and clinically disruptive adverse effects associated with antipsychotic treatment. Structured assessments consistently report prevalence rates exceeding 40–70%, whereas spontaneous reporting remains substantially lower, reflecting persistent under-recognition in routine clinical practice [5]. Beyond its impact on quality of life, SD is strongly associated with reduced adherence, treatment discontinuation, and impaired relational functioning, ultimately compromising long-term outcomes [4,8,24,25].

Antipsychotic-related SD is multifactorial. Dopamine D2 receptor blockade and consequent hyperprolactinemia play central roles, particularly in decreased libido, arousal impairment, and orgasmic dysfunction [8,9,26]. However, prolactin-independent mechanisms—including serotonergic, adrenergic, cholinergic, and nitric oxide pathways—also contribute, underscoring the complexity of pharmacologically induced SD [27].

Naturalistic comparative evidence demonstrates substantial variability in sexual tolerability across antipsychotic agents. Earlier comparative studies suggested clinically meaningful differences in sexual adverse effects between antipsychotics with distinct dopaminergic profiles, such as clozapine and haloperidol [28]. Similar pharmacologically mediated sexual adverse effects have also been described across other psychotropic drug classes, highlighting the broader neurobiological vulnerability of sexual functioning to pharmacological modulation [29]. Systematic reviews evaluating pharmacological interventions for antipsychotic-induced sexual dysfunction further support targeted therapeutic strategies for managing these adverse effects [30]. Prolactin-elevating agents such as risperidone are consistently associated with higher rates of SD, whereas prolactin-sparing compounds show more favorable profiles [31]. Switching strategies have therefore emerged as a clinically relevant approach when sexual adverse effects compromise adherence. Previous studies have shown that switching to agents such as quetiapine, ziprasidone, or aripiprazole is associated with clinically meaningful improvements in sexual functioning [20,21,23,32,33] findings supported by meta-analytic evidence [34,35]. Overall, cariprazine has demonstrated a favorable tolerability profile [36].

Within this framework, the present study provides prospective real-world evidence specifically focused on cariprazine. In patients switched due to sexual dysfunction, we observed large and clinically meaningful improvements across all SALSEX domains, accompanied by significant reductions in prolactin levels. The magnitude and consistency of these changes are comparable to those reported with other partial dopamine agonists, supporting the validity of prolactin-sparing strategies in clinical practice.

Importantly, improvement was not restricted to a single domain but involved a global restoration of sexual functioning. The pattern of change—early improvement in libido and arousal followed by more gradual recovery of orgasm-related domains—is clinically plausible and consistent with patient-reported trajectories in routine care.

Although prolactin reduction provides a biologically plausible mechanism, the absence of a direct correlation between hormonal changes and sexual outcomes suggests that additional pharmacological factors may be involved. Cariprazine’s preferential D3 receptor affinity may contribute to modulation of reward and motivational processes relevant to sexual function [17]. However, as with other partial dopamine agonists, rare cases of impulse-control symptoms, including hypersexuality, have been reported [37,38], highlighting the complexity of dopaminergic modulation.

Sexual improvement was not associated with changes in psychiatric symptom severity, suggesting that the observed effects are not merely secondary to clinical stabilization. This finding is clinically relevant, as it supports the feasibility of switching antipsychotic treatment to improve tolerability without compromising psychiatric control.

Treatment persistence was high, and no discontinuations were attributed to sexual adverse effects. These findings reinforce the clinical acceptability of cariprazine, particularly in patients for whom sexual tolerability is a priority.

From a clinical perspective, our results support the systematic assessment of sexual function in patients receiving antipsychotic treatment. Structured instruments such as SALSEX facilitate detection of underreported adverse effects and may guide treatment optimization [39]. Addressing sexual dysfunction early is particularly relevant in the context of long-term adherence and patient-centered care. Sexual adverse effects remain underreported, and recruitment may be highly limited by the sensitive nature of the topic and variability in clinical reporting practices. These challenges have also been documented in previous research evaluating pharmacological interventions for antipsychotic-induced sexual dysfunction. The REMEDY trial, a randomized controlled study designed to evaluate treatment strategies for antipsychotic-related sexual dysfunction, had to be discontinued after approximately one year due to insufficient patient recruitment, illustrating the considerable practical difficulties of conducting prospective research in this area [40]. Finally, addressing sexual dysfunction early may represent not only a strategy to enhance tolerability but also a key component of comprehensive patient-centered care in psychosis [41].

### 4.1. Limitations

This study has several limitations that should be acknowledged. First, the modest sample size reduces statistical power for subgroup and multivariate analyses and may limit detection of subtle interindividual variability. Second, the observational open-label design without a control group introduces potential expectancy effects and limits causal inference. Although the consistency of findings across multiple domains strengthens internal coherence, the possibility of regression to the mean cannot be entirely excluded.

Endocrine measurements were obtained as part of routine clinical practice rather than through a strictly protocol-mandated schedule, which may introduce variability in timing and laboratory conditions. Additionally, analyses were conducted using complete-case data without formal imputation procedures, which may increase susceptibility to attrition-related bias.

Given the exploratory nature of several secondary analyses, adjustments for multiple comparisons were not systematically applied; therefore, findings should be interpreted cautiously and considered hypothesis-generating. Finally, the relatively short follow-up period precludes conclusions regarding the long-term sustainability of sexual improvement and endocrine stability.

### 4.2. Future Directions

Future research should aim to confirm these findings in larger prospectively designed cohorts with sufficient statistical power to explore mechanistic pathways in greater depth. Studies incorporating longitudinal hormonal profiling, including dynamic prolactin measurements and broader endocrine panels, may help clarify whether sexual recovery reflects endocrine threshold effects or more complex dopaminergic modulation. Randomized controlled trials comparing different prolactin-sparing strategies would also be valuable to determine whether the observed improvement is specific to cariprazine or generalizable across partial dopamine agonists. Finally, integrating patient-reported outcome measures and quality-of-life indices may further elucidate the functional and psychosocial impact of sexual recovery in real-world psychiatric populations.

## 5. Conclusions

Switching to cariprazine was associated with clinically meaningful improvement in sexual function. Improvements occurred across multiple domains and were independent of symptom severity changes. Prolactin reduction contributed but did not fully explain the observed effects, whereas cariprazine represents a viable prolactin-sparing strategy in clinical practice.

## Figures and Tables

**Table 1:** Baseline characteristics.

Variable	Group A (De Novo)	Group B (Switch)
*n*	14	28
Age (years)	31.86 ± 7.67	38.58 ± 12.92
Female (%)	28.6%	39.3%
SALSEX total	2.79 ± 2.01	8.04 ± 2.76
CGI-S sexual	2.50 ± 0.94	4.14 ± 0.97
CGI-S psychosis	4.36 ± 1.15	3.29 ± 1.46
Prolactin (ng/mL)	14.89 ± 14.12	42.07 ± 59.22

**Table 2:** Change in SALSEX total score.

Group	Baseline	Month 1	Month 3	*p*-Value	Cohen’s d
A (de novo)	2.79 ± 2.01	1.64 ± 1.34	1.23 ± 1.24	0.023	0.93
B (switch)	8.04 ± 2.76	4.39 ± 2.87	2.41 ± 2.06	<0.001	2.27

**Table 3:** SALSEX domain scores.

Domain	Group	Baseline	Month 3	*p*-Value	Cohen’s d
Libido	A	0.79	0.38	0.062	0.75
Libido	B	1.57	0.50	<0.001	1.51
Orgasm delay	B	1.79	0.55	<0.001	1.51
Anorgasmia	B	1.29	0.55	<0.001	1.24
Arousal	B	1.64	0.36	<0.001	2.12
Tolerability	B	1.75	0.45	<0.001	2.19

**Table 4:** Hormonal changes.

Variable	Group	Baseline	Month 3	Δ	*p*-Value
Prolactin	A	14.91	13.66	−1.25	0.76
Prolactin	B	42.07	29.52	−12.56	0.01

**Table 5:** Treatment outcomes.

Group	Continued	Discontinued (AE)	Discontinued (Inefficacy)
A	13	1	0
B	22	1	1

**Table 6:** Change in BPRS Total Score from Month 1 to Month 3.

Group	*n*	Month 1 Mean ± SD	Month 3 Mean ± SD	Δ Mean	*p*-Value	Cohen’s d
A (de novo)	14	42.36 ± 21.44	23.21 ± 11.29	−19.14	0.004	1.05
B (switch)	28	62.36 ± 17.54	29.29 ± 10.56	−33.07	<0.001	1.93

## Data Availability

Data are available from the corresponding author upon reasonable request, subject to institutional and privacy restrictions.
